# Posterior mediastinal ganglioneuroblastoma in an adolescent: A case report and review

**DOI:** 10.1111/1759-7714.13277

**Published:** 2019-12-14

**Authors:** Nodoka Sekiguchi, Takuro Noguchi, Toshirou Fukushima, Takashi Kobayashi, Takesumi Ozawa, Yoshinori Sato, Tetsu Takeda, Kazuo Yoshida, Tomonobu Koizumi

**Affiliations:** ^1^ Department of Comprehensive Cancer Therapy Shinshu University School of Medicine Matsumoto Japan; ^2^ Department of Laboratory Medicine Shinshu University School of Medicine Matsumoto Japan; ^3^ Division of Thoracic Surgery Suwa Red Cross Hospital Suwa Japan

**Keywords:** Bone metastasis, mediastinal tumor, neuroblastoma, posterior mediastinum

## Abstract

Ganglioneuroblastoma is an uncommon malignant tumor of the sympathetic nervous system, which is considered a disease of children with the majority of cases in patients less than four years old and it rarely occurs in adults. We encountered a very unusual case of a posterior mediastinal ganglioneuroblastoma that developed in a 17‐year‐old male adolescent who underwent successful excision of the mediastinal mass and remained stable postoperatively. However, he developed lumbago one year after the surgery. Radiographic findings revealed osteolytic lesions in the lumbar vertebra and histological analysis confirmed bone metastasis of ganglioneuroblastoma. Here, we report the clinical course and present a review of the literature regarding adolescent and adult onset mediastinal ganglioneuroblastoma.

## Introduction

Ganglioneuroblastoma (GNB) is an uncommon malignant tumor of the adrenal gland or sympathetic nervous system. GNB has two histological features of ganglioneuroma (benign) and neuroblastoma (malignant), thus showing malignant or potentially malignant behavior.^1^, ^2^ GNB is most common in children, especially age 1–2 years, with a median age at diagnosis of 22 months; most cases are diagnosed by 10 years of age.^2^, ^3^, ^4^, ^5^ In addition, the most common sites of origin are the adrenal medulla (about 35%), extraadrenal retroperitoneum (30%–35%), and posterior mediastinum (20%).^4^ Although cases of adolescent or adult onset GNB have been reported in the literature, they are extremely rare.^6^, ^7^, ^8^, ^9^, ^10^, ^11^, ^12^, ^13^ With regard to mediastinal GNBs, less than 50 cases of adolescent or adult mediastinal GNB have been reported to date.^6^, ^7^, ^8^, ^9^, ^10^ We report a case of posterior mediastinal GNB in a 17‐year‐old male adolescent. The tumor was detected by chest radiography and radical tumor resection was performed. However, multiple bone metastases developed one year after surgery. Here, we present the clinical course and a review of adult and/or adolescent onset mediastinal GNB.

## Case report

**Figure 1:**
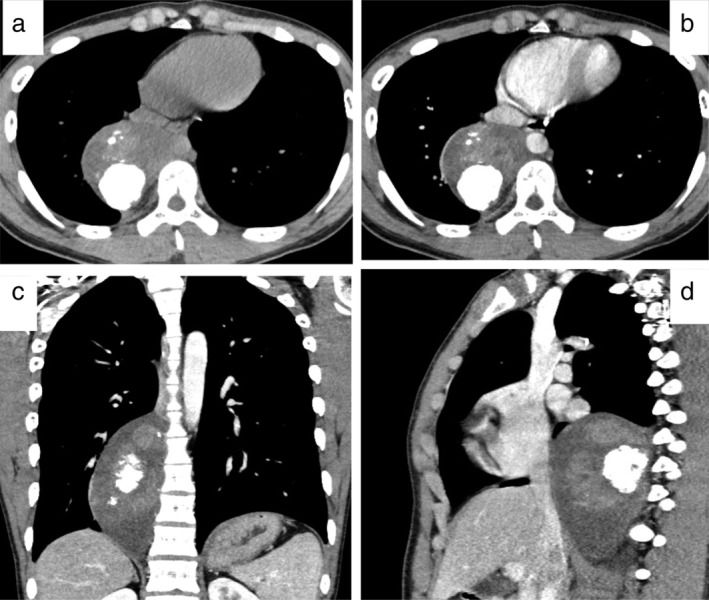
(**a**) Chest computed tomography showed a right‐sided posterior mediastinal mass with aggregated and nodular calcification. (**b**) There were enhanced areas within the mass on axial contrast computed tomography. (**c** and **d**) Coronal computed tomography showed that the tumor extended along the right vertebral area.

**Figure 2:**
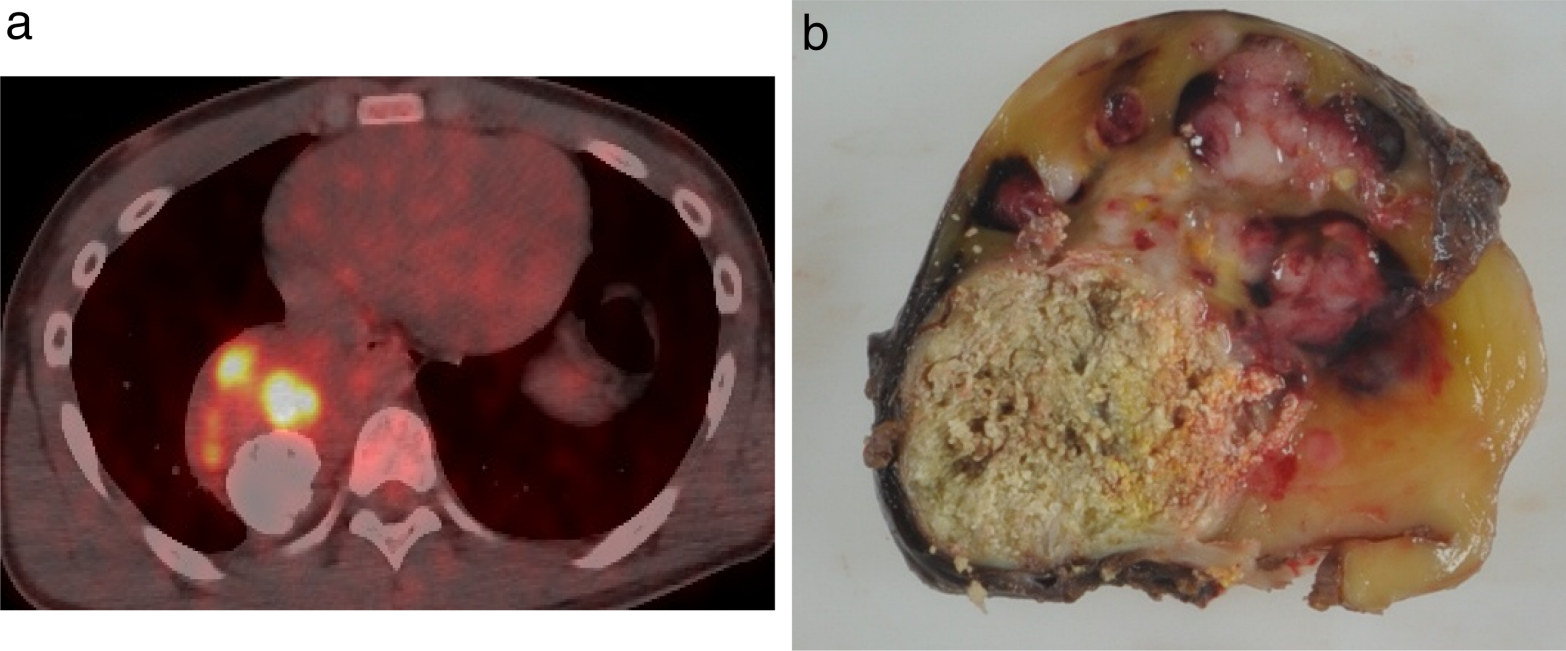
(**a**) Positron emission tomography with fluorodeoxyglucose‐computed tomography (FDG‐PET/CT) showed areas of increased FDG uptake within the tumor. (**b**) Macroscopic section of the resected tumor. The mass measured 13.5 × 7.5 × 6 cm and was variegated tan‐yellow in appearance with aggregated calcification and focal necrosis. Raised, dome‐shaped, white‐pink irregular nodules were seen inside the capsule.

**Figure 3:**
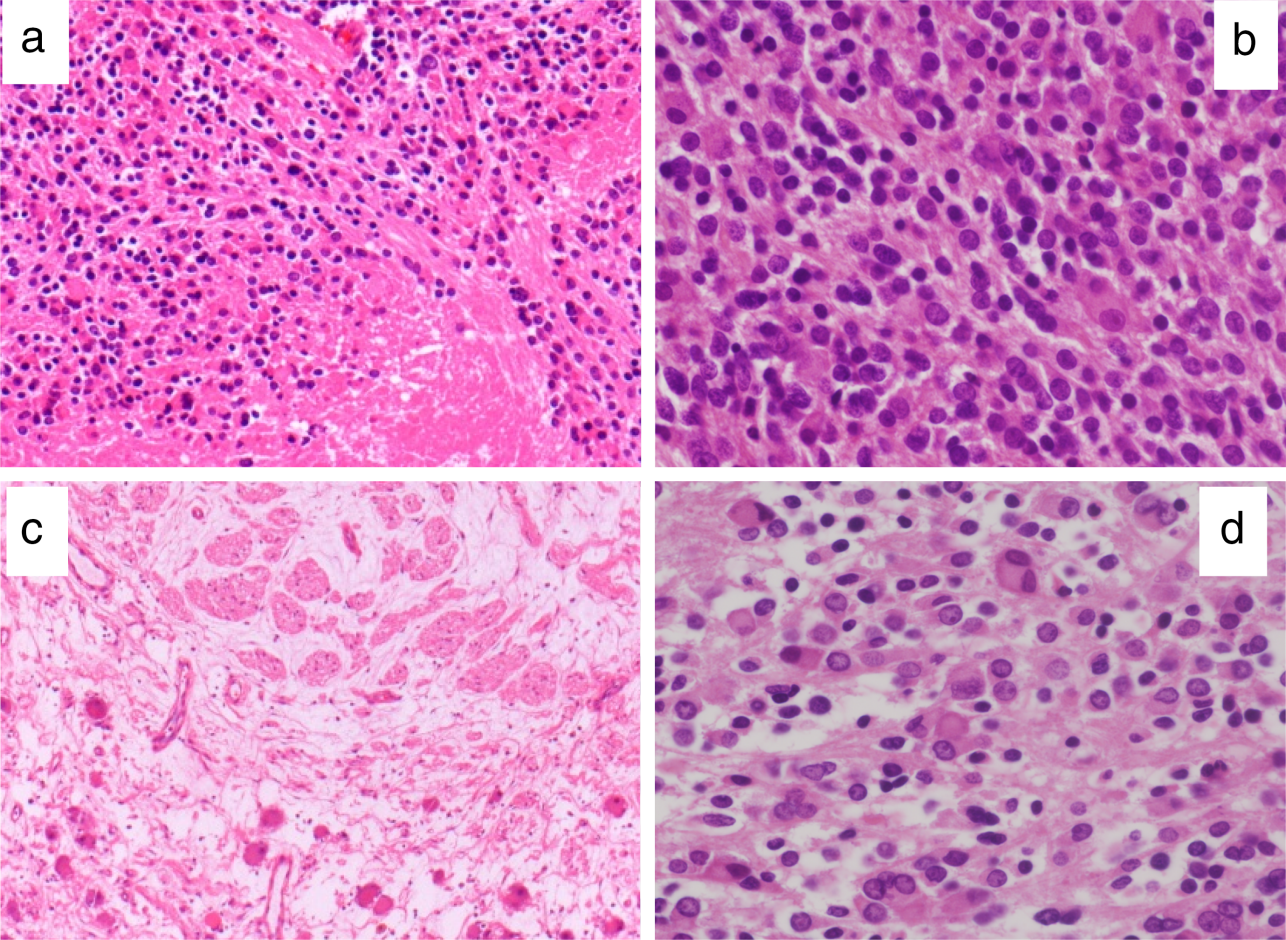
The pathological findings of the resected mass indicated grossly visible neuroblastomatous nodules with ganglioneuromatous components. (**a** and **b**) In the neuroblastomatous area, small oval cells were mainly observed with increased mitosis or geographic necrosis. (**c** and **d**) In contrast, predominantly mature ganglion cells and gangioneuromatous elements were observed in myxoid and fibrous stroma (**a**, HE ×40, **b**, HE ×100, **c**, HE ×40, **d**, HE ×100).

A 17‐year‐old male adolescent was admitted to hospital with an abnormal shadow detected on chest radiography in a school survey. He had no relevant past history, including his birth and development. He had been asymptomatic and physical examination revealed no abnormal findings. Chest radiography and computed tomography (CT) (Fig 1) revealed a giant posterior mediastinal tumor with aggregated and nodular calcification. Histological findings by percutaneous needle biopsy revealed neuroblastoma with primitive undifferentiated cells and ganglioneuroma with mature ganglion cells and Schwann cells, suggesting GNB. Positron emission tomography with fluorodeoxyglucose‐computed tomography (FDG‐PET/CT) revealed positive FDG uptake areas in the mediastinal mass lesion that were enhanced on contrast CT (Fig 2a) and no abnormal FDG uptake in other organs. Laboratory findings showed that neuron‐specific enolase (NSE) was 17.9 ng/mL (normal < 16.7 ng/mL), and urine vanillylmandelic acid (VMA) and homovanillic acid (HVA) levels were 5.0 mg/day (normal < 4.9 mg/day) and 7.4 mg/day (normal < 6.5 mg/day), respectively. Thoracic surgery was performed for the mediastinal tumor and the mass was removed successfully. The tumor was variegated tan‐yellow with focal necrosis and measured 13.5 × 7.5 × 6 cm (Fig 2b). The pathological findings of the resected mass contained grossly visible neuroblastomatous nodules including ganglioneuromatous components. In the neuroblastoma area, mainly small oval cells were observed with increased mitosis or geographic necrosis (Fig 3a,b). Immunohistochemical staining revealed tumor cells positive for chromogranin A, synaptophysin, S‐100, and NSE. Ki‐67 labeling index was 25% in the area. In contrast, predominantly mature ganglion cells and gangioneuromatous elements were observed in myxoid and fibrous stroma (Fig 3c,d). Immunohistochemical staining revealed these cells positive for chromogranin A, synaptophysin, and NSE. S‐100 was negative for these cells. Ki‐67 labeling was less than 1%. Based on these histological findings, the diagnosis of GNB was made. The present case showed no *MYCN* oncogene amplification.

**Figure 4:**
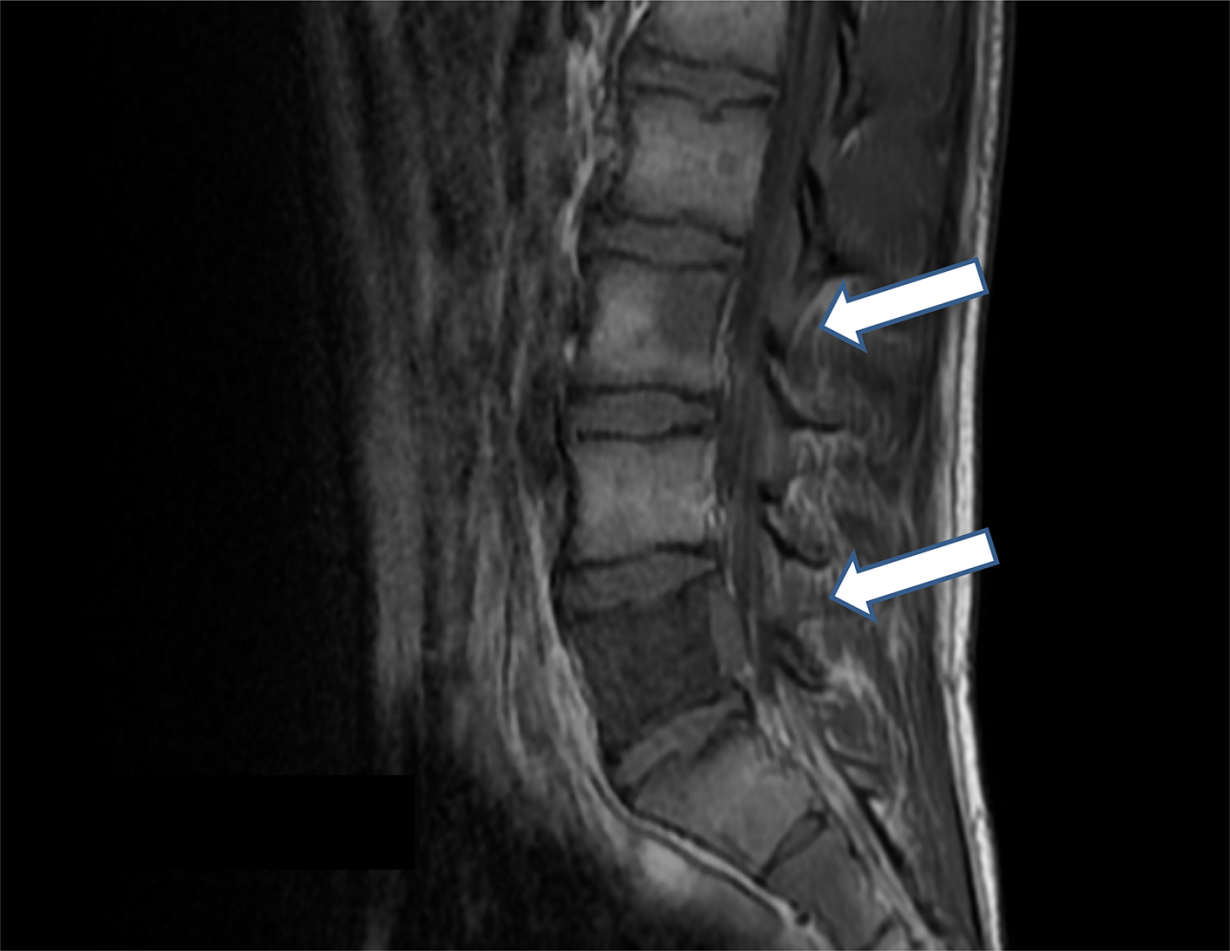
Magnetic resonance imaging showed osteolytic lesions in L3 and L5, suggesting bone metastases.

Serial whole CT and iodine‐123‐metaiodobenzylguanidine (^123^I‐MIBG) examination were performed every six months after surgery and no abnormal findings were detected. However, he presented with lumbago one year after surgery. Magnetic resonance imaging (MRI) showed multiple osteolytic bone metastases, especially in the lumbar vertebrae (L3 and L5) (Fig 4). FDG‐PET/CT revealed increased FDG uptake including the lumbar vertebrae, iliac and rib bone, suggesting multiple bone metastases. There were no other findings of abnormal FDG uptake except these bone lesions. Needle biopsy from the lumbar spine (L5) was performed and the histological findings confirmed metastatic GNB from the posterior mediastinal tumor. NSE was normal (13.7 ng/mL) but urine VMA (11.9 mg/day) and HVA (7.4 mg/day) were increased. Radiotherapy (3 Gy × 10 fractions, total 30 Gy) to the metastatic lesions (L5) was performed, which relieved pain. Subsequently, the patient received four cycles of combined chemotherapy with cisplatin, cyclophosphamide vincristine, and doxorubicin every three to four weeks. In addition, administration of denosumab (a fully human monoclonal antibody that inhibits receptor activator of nuclear factor‐kappa B ligand) was continued every four weeks.

The patient again developed lumbago again and palliative radiotherapy (3 Gy × 10 fractions, total 30 Gy) was added for bone metastasis (L3). The patient is currently alive, two years after the initial recurrence.

## Discussion

Here, we present the clinical course of mediastinal GNB discovered in a 17‐year‐old male adolescent. As GNBs occur almost exclusively in the pediatric population,^2^, ^3^, ^4^, ^5^ adult onset GNB is uncommon and the mediastinum as the primary site is a further rare clinical manifestation.^6^, ^7^, ^8^, ^9^, ^10^, ^11^, ^12^, ^13^ These points are noteworthy in the present case.

Adam and Hochholzer^7^ presented a summary of 80 cases of GNB of the posterior mediastinum treated between 1944 and 1978, and reported that among 10 (12.5%) of these patients were adolescents (12–20 years old) and three (3.8%) were adults (over 20 years old). Jrebi *et al*.^8^ reviewed 15 cases of adolescent and adult neuroblastoma and/or GNBs in their institute and only one of these cases involved the mediastinum. Subsequently, Mizuno *et al*.^10^ reviewed the literature and found 49 cases of adult onset GNB by 2010, including their own case. They reported posterior mediastinal GNBs in eight adult patients (16.7%). Furthermore, we searched the PubMed database for papers published after 2010 using the keywords “ganglioneuroblastoma” “adult” “adolescent” and “mediastinum.” We found an additional few cases of adolescent or adult mediastinal GNB.^14^, ^15^ Thus, less than 50 cases have been reported in the English literatures. Therefore, mediastinal GNBs in adults or adolescents are extremely rare clinical manifestations and we should take into consideration adult GNBs as posterior mediastinal tumors.

CT and MRI are the most commonly used imaging modalities for assessment of GNBs.^4^, ^15^ The features of GNBs are variable on enhanced CT, ranging from well‐marginated, oblong paravertebral masses with homogeneous enhancement to irregular, cystic, hemorrhagic, or locally invasive masses.^4^, ^15^ Approximately 50% of thoracic neuroblastomas/GNBs have coarse, finely‐stippled or curvilinear calcifications.^15^ On MRI, neuroblastomas and GNBs are typically heterogeneous with variable enhancement and high signal intensity on T1W1 in hemorrhagic areas and hyperintense signals on T2W1 in cystic lesions.^4^, ^15^

Early and radical surgery is the best treatment available for mediastinal GNBs.^5^, ^6^, ^7^, ^8^, ^9^, ^10^, ^11^ The prognosis is generally favorable with a two‐ and five‐year survival rates of 92% and 88%, respectively, reported in a large series.^7^ However, Adam and Hochholzer^7^ showed that recurrence in posterior mediastinal GNBs after surgery occurred only in adult/adolescent patients. In general, older children with neuroblastoma/GNB had poorer outcome than younger children.^3^, ^5^, ^16^ In addition, tumor size at diagnosis may be related to the prognosis in adult GNB. Adult GNB tumors >8 cm in diameter arising from the retrospective cavity tend to metastasize to other distant organs.^10^ The size of the lesion in the present case (13 cm) was large compared to those in other reports of mediastinal GNB,^7^, ^9^, ^10^, ^11^ and therefore the risk of recurrence in our case may have been increased at the time of diagnosis. Therefore, cases of adolescent/adult GNB should be followed‐up carefully even after complete resection.

The present case developed bone metastasis, which is the most common site for metastases in patients with GNB.^4^, ^15^, ^17^ Cortical metastasis may lead to diffuse bone marrow invasion, resulting in reduced responsiveness to chemotherapy and poor prognosis.^4^, ^5^, ^12^ Hepatic metastases are also common and other less common areas of metastasis include the lungs and brain.^4^

Information about the role of chemotherapy and optional therapeutic agents in GNB is limited. Drugs used in pediatric patients are cyclophosphamide vincristine, doxorubicin, and combinations with platinum and etoposide. Raina *et al*.^18^ reported a case of GNB that showed an excellent response to such combined chemotherapies. However, little information is available regarding the usefulness of chemotherapy for advanced or metastatic GNB.^5^, ^6^, ^19^ In addition, several case series studies have suggested the usefulness of neoadjuvant chemotherapy in patients with neuroblastoma/GNB,^5^, ^6^, ^18^, ^19^, ^20^ but the effects on prognosis remain uncertain because of the rarity of the disease. In the present case, four cycles of chemotherapy including cisplatin, cyclophosphamide vincristine, and doxorubicin were performed, but the effects could not be evaluated because of the lack of target lesions in this case. Additional clinical experience is required to evaluate the efficacy of chemotherapy in this disease.

In summary, we describe a rare case of adolescent posterior mediastinum GNB with the development of bone metastasis one year after radical thoracotomy. The observations in this case suggest that GNB should be considered to be a posterior mediastinal tumor even in adult patients. Based on our experience in this case, we emphasize that long‐term follow‐up and/or careful examination are necessary in patients with GNB, especially with a large‐sized tumor.

## Disclosure

None of the authors have any potential conflicts of interest.
